# Postural analysis reveals persistent changes in paper wasp foundress behavioral state after conspecific challenge

**DOI:** 10.1002/ece3.10436

**Published:** 2023-08-31

**Authors:** Andrew W. Legan, Caleb C. Vogt, Michael J. Sheehan

**Affiliations:** ^1^ Laboratory for Animal Social Evolution and Recognition, Department of Neurobiology and Behavior Cornell University Ithaca New York USA; ^2^ Department of Entomology University of Arizona Tucson Arizona USA

**Keywords:** automated tracking, behavioral plasticity, field study, pose estimation, social insect

## Abstract

Vigilant animals detect and respond to threats in the environment, often changing posture and movement patterns. Vigilance is modulated not only by predators but also by conspecific threats. In social animals, precisely how conspecific threats alter vigilance behavior over time is relevant to long‐standing hypotheses about social plasticity. We report persistent effects of a simulated conspecific challenge on behavior of wild northern paper wasp foundresses, *Polistes fuscatus*. During the founding phase of the colony cycle, conspecific wasps can usurp nests from the resident foundress, representing a severe threat. We used automated tracking to monitor the movement and posture of *P. fuscatus* foundresses in response to simulated intrusions. Wasps displayed increased movement, greater bilateral wing extension, and reduced antennal separation after the threat was removed. These changes were not observed after presentation with a wooden dowel. By rapidly adjusting individual behavior after fending off an intruder, paper wasp foundresses might invest in surveillance of potential threats, even when such threats are no longer immediately present. The prolonged vigilance‐like behavioral state observed here is relevant to plasticity of social recognition processes in paper wasps.

## Graphical abstract

This study set out to address the question: how does persistent vigilance manifest in individual movement and posture in paper wasps? We simulated intruder encounters and used automated tracking to analyze movement and posture in wild *Polistes fuscatus* foundresses. Wasps displayed increased vigilance after the threat was removed, in the form of increased movement, greater bilateral wing extension, and reduced antennal separation.

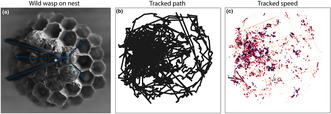

## 1 INTRODUCTION

Vigilance behavior in animals is demonstrated by changes in movement and body posture, as famously exemplified by the still, bipedal stance of meerkat sentinels (Santema & Clutton‐Brock, 2013). Movement and posture of specific body parts, especially the head and sensory organs, are responsible for vigilance quality because they directly influence perception. For example, chaffinches turn their heads more after seeing a cat (Jones et al., 2007) and vigilant baboons blink less (Matsumoto‐Oda et al., 2018). Sometimes animals must sacrifice vigilance quality in favor of other important activities. For example, juncos forfeit some vigilance quality to lower their heads and eat (Lima & Bednekoff, 1999).

Social animals, though characterized by their cooperative associations, face threats posed by conspecifics (Abbot, 2022). Recognition is an important mechanism mediating intraspecific aggression because encounters with different types of individuals can impact fitness in distinct ways (Bourke, 2011; Gherardi et al., 2012; Leonhardt et al., 2016; Mateo, 2004; Sheehan & Bergman, 2016). Social insects exhibit plasticity in nest‐guarding behavior in response to the frequency and valence of interactions with nestmates and non‐nestmates (Fürst et al., 2011; Liebert & Starks, 2004; Mora‐Kepfer, 2014; Starks et al., 1998). In response to encounters with non‐nestmates, honeybees restrict admittance to the colony, sometimes rejecting their own nestmates (Couvillon et al., 2008; Downs & Ratnieks, 2000). Signal detection theory predicts these rejection errors result from a restricted acceptance threshold (Reeve, 1989; Wiley, 2013). With more frequent intruder encounters, the cost of a permissive acceptance threshold increases. To account for this, the acceptance threshold is reduced to minimize erroneous acceptance of non‐nestmates, with the side‐effect of increasing erroneous rejection of nestmates. An alternative view considers variation in recognition behavior in terms of investment in recognition accuracy (Sheehan & Reeve, 2020). Recognition accuracy can be improved by persistent vigilance behavior of nest guards. Shifts in vigilance at the group level have been documented in honey bees, which allocate more guards at the colony entrance in response to threats (Breed et al., 1992; Downs & Ratnieks, 2000). How persistent vigilance manifests in individual posture is not well characterized in social insects.

Recent advances in computer vision have made automated tracking software publicly available for application to postural analysis of animal behavior. Such methods have been applied to study neurobiological mechanisms of animal posture, movement, collective behavior, and social interactions (Crall et al., 2018; Dell et al., 2014; Mathis & Mathis, 2020; Wang et al., 2022). Many animal behaviors are robust to laboratory conditions and can be studied in a controlled environment. For example, automated tracking has been used to characterize the foraging behavior of hawkmoths *Manduca sexta* (Dahake et al., 2018; Deora et al., 2021) and to characterize the wing kinematics of flies and honey bees, as well as honey bee wing fanning behavior (Altshuler et al., 2005; Muijres et al., 2014; Peters et al., 2017). Complex social behaviors are less robust to laboratory conditions, requiring field observations to draw reliable conclusions. However, few studies have applied automated tracking of individual social animal posture in the wild (but see Peters et al., 2017).

*Polistes* paper wasps are ideal for field studies of vigilance behavior. Compared to eusocial ants, honey bees, and hornets, *Polistes* societies remain relatively small, with up to ~135 nest cells (Reeve, 1991). Nests are generally single‐layer, allowing them to be filmed in their entirety with one camera. *Polistes* nests are often founded by single individuals. Regarding automated tracking of animal posture, single individuals are easier to track than multiple, unmarked individuals.

This study set out to address the question: how might persistent vigilance manifest in individual movement and posture in paper wasps? *Polistes* foundresses guard the nest from conspecific intruders which can rob their brood or usurp their nests (Gamboa et al., 1992; Kasuya et al., 1980; Miller et al., 2018; Reeve, 1991; Sakagami & Fukushima, 1957; Sheehan et al., 2015). Automated tracking of wild *Polistes* foundress behavior is an as‐yet unapplied tool for understanding the effects of intruder encounters on vigilance. We simulated intruder encounters and used automated tracking to analyze movement and posture of wild *Polistes fuscatus* foundresses.

## 2 MATERIALS AND METHODS

We studied solitary *P. fuscatus* foundresses (hereafter “foundresses”) on their nests at the Liddell Field Station in Ithaca, NY (42°27′36.7″ N, 76°26′39.2″ W). In the spring of 2020, wild wasps initiated nests in modified wooden bird boxes (11.5 cm × 12.5 cm × 13.5 cm). All experiments were carried out from July 4 to July 9, 2020, before workers emerged. Experiments were carried out between 2:00 PM and 8:00 PM EST, during the wasps' active phase in peak summer. The mean nest size was 33 ± 8 (SD) cells. The experimental apparatus consisted of a 162.5 cm wooden dowel (7 mm diameter) guided through a 122 cm metal cylinder (1 cm diameter), taped to a step ladder. The assays were video‐recorded from below using a tripod‐mounted Nikon D7200 camera with a Sigma Macro HSM lens with an optical stabilizer (focal length: 105 mm; aperture: f/2.8).

On the morning of July 4th, 2020, intruder wasps were collected from nests at a site (42°24′57.6″ N, 76°31′22.6″ W) 8.15 km southwest of the Liddell Station. They were housed individually in deli cups and provided a sugar cube and cotton‐stopped water vial until the time of the experiment. These wasps were also nest foundresses but are simply referred to as “intruders” in the text for clarity. Since *P. fuscatus* foundresses often cooperate with related individuals to co‐found nests, it was important that the intruders were not closely related to the foundresses. Foraging and dispersal distances of *P. fuscatus* are estimated to be on the order of hundreds of meters (Bluher et al., 2020; Dew & Michener, 1978). Therefore, we are confident that foundresses were not closely related to intruders and had not previously encountered them. Foundresses were size matched to intruders within 0.028 ± 0.013 grams (SD). Immediately before each simulated intruder trial, the intruder was freeze‐killed and fixed to a wooden dowel using an insect pin. Unique intruders were presented as the stimulus in each simulated intruder trial. On a different day, each foundress was presented with the wooden dowel alone. The order of stimulus presentations (intruder or wooden dowel) was random, with half of the foundresses presented with the dowel on the first day and half presented with the intruder on the first day (Table 1). The amount of time between the two presentations (intruder or wooden dowel) ranged from 2 to 5 days.

**TABLE 1:** Metadata, summary tracking performance, and measures of movement and posture for tracked intervals of behavioral assays.

Trial_ID	Interval	Treatment	Nest_ID	Head_percent_frames_tracked	Thorax_percent_frames_tracked	Abdomen_percent_frames_tracked	LWing_percent_frames_tracked	RWing_percent_frames_tracked	LAnt_percent_frames_tracked	RAnt_percent_frames_tracked	Distance_Head_Traveled	Distance_Thorax_Traveled	Mean _Wing_degrees	Mean_Ant_Degrees
8565	1	Intruder	12	100	100	100	97.95	100	52.45	41.8	13,730	14,886	5.2	106.2
8565	3	Intruder	12	99.44	99.75	95.08	84.74	84.23	84.59	85.34	45,709	44,694	40	105.3
8569	1	Dowel	22	82.91	86.11	83.47	76.57	82.19	78.33	78.95	22,312	20,821	24.5	113.4
8569	2	Dowel	22	96.95	97.47	96.12	95.62	95.76	91.76	92.8	27,419	26,645	29.7	115.3
8569	3	Dowel	22	88.07	88.24	87.97	86.67	87.75	82.87	80.95	21,724	17,653	22	119.2
8571	1	Dowel	19	56.53	56.86	54.65	47.4	52.76	48.05	47.28	19,081	16,716	16.7	107.9
8571	2	Dowel	19	81.59	78.21	73.14	71.38	70.87	77.5	75.35	23,856	16,449	17.7	118.2
8571	3	Dowel	19	99.95	99.95	99.2	99.14	99.55	97.29	97.37	16,086	18,834	19.8	116.2
8572	1	Intruder	7	99.51	98.02	65.53	94.6	95.36	92.06	88.5	24,982	16,631	9.3	104.5
8572	3	Intruder	7	90.65	88.56	75.62	71.45	75.22	70.41	76.04	162,031	104,254	43.5	97.3
8573	1	Intruder	15	99.76	99.77	98.71	80.81	99.67	95.07	92.27	33,705	27,853	17.4	103.8
8573	3	Intruder	15	78.22	84.61	69.33	62.85	68.91	41.71	52.31	90,437	78,104	40.5	99.8
8574	1	Dowel	60	99.95	99.95	99.95	99.95	99.95	99.65	99.89	7437	4977	6.8	114.6
8574	2	Dowel	60	52.88	51.9	47.23	50.1	37.17	41.08	46.29	11,682	9452	14.1	93.6
8574	3	Dowel	60	24.2	9.43	6.4	5.31	5.14	21.66	6.41	11,720	6882	23	97.7
8601	1	Dowel	12	99.91	99.94	95.74	99.47	97.3	96.5	94.52	23,130	23,804	27.1	110.6
8601	2	Dowel	12	99.88	99.92	98.51	99.09	99.29	98.34	94.71	15,964	12,925	32.5	118.1
8601	3	Dowel	12	98.98	98.89	97.28	96.83	97.11	93.52	96.46	18,242	17,386	19.6	112.1
8604	1	Intruder	22	95.26	96.3	94.1	92.19	91.95	75.73	79.98	39,618	28,317	16.7	111.7
8604	3	Intruder	22	98.53	98.78	95.74	92.16	93.94	78.81	80.73	47,008	36,419	35.3	99.1
8605	1	Dowel	15	97.61	98.75	97.96	94.66	95.52	92.59	92.46	17,129	12,871	8	115.5
8605	2	Dowel	15	95.5	94.83	90.39	88.57	87.29	87.51	88.46	43,088	36,797	30.5	98.6
8605	3	Dowel	15	99.03	99.3	98.48	89.15	79.54	87.3	94.63	22,119	20,913	32.9	104.1
8607	1	Dowel	7	96.13	94.71	90.65	83.64	87.23	84.44	84.53	30,114	20,307	8.6	100.9
8607	2	Dowel	7	99.97	99.85	97.74	98.3	95.56	93.32	88.11	49,368	33,301	29.7	100.5
8607	3	Dowel	7	99.17	99.2	98.47	95.4	97.79	93.7	94.8	26,508	17,358	12.5	103.4
8608	1	Intruder	19	95.37	94.39	50.09	47.52	49.74	63.23	39.61	34,940	25,577	17.6	111.9
8608	3	Intruder	19	99.14	99.17	95.99	93.33	98.47	79.99	81.35	54,902	40,994	37.2	101.1
8609	1	Intruder	60	99.26	99.29	94.83	94.21	98.38	93.17	93.32	5859	5844	21.7	112
8609	3	Intruder	60	95.47	94.69	79.09	87.82	86.77	77.14	79.06	44,809	27,604	32.8	101.8

**VIDEO 1:** Sequence shows 250 frame excerpts of videos of all wasps assayed before, during, and after dowel and simulated intruder presentations, respectively.

**VIDEO 2:** Representative video of the foundress from nest box 12 before, during, and after a simulated intruder encounter.

Foundresses were presented with an intruder for slightly more than 5 min (320 s) to simulate an extreme threat, such as nest usurpation (Gamboa et al., 1992). All assays consisted of three 320‐second intervals: pre‐stimulus, stimulus, and post‐stimulus. A time interval of 320 s was chosen to balance three needs. First, we aimed to attempt automated tracking and compare postural dynamics between each experimental interval and thus made all intervals equal in duration. Second, we sought to record foundress behavior for at least 5 min before and after stimulus presentation to capture the baseline behavior and stimulus‐induced behavior of foundresses. Third, we intended to simulate a severe threat to the foundress, similar to a prolonged nest usurpation event, with the aim of inducing heightened vigilance and estimating postural correlates of vigilance. *P. fuscatus* cofoundresses that successfully guarded their nests repelled intruders within 40 s (Gamboa et al., 1992). We chose a longer time interval for intruder encounters since we were studying single‐foundress nests which might be more vulnerable to nest usurpation.

All nests were undisturbed, with experimental apparatus in place, for at least 5 min before beginning the pre‐stimulus interval. During the stimulus presentation in both simulated intruder and wooden dowel trials, the stimulus was moved slightly by the experimenter at one‐minute intervals to animate the stimulus. Three foundresses were excluded from analysis because a live intruder visited the nest during the experiment, and one foundress was excluded from analysis because the foundress was accidentally flushed from the nest while setting up the experimental apparatus. Ultimately, six intruder assays and six control assays were analyzed. While smaller than intended, this sample size was adequate for statistical analyses given the large estimated effect sizes of the intruder‐induced persistent changes in foundress posture (see Section 3).

**FIGURE 1:**
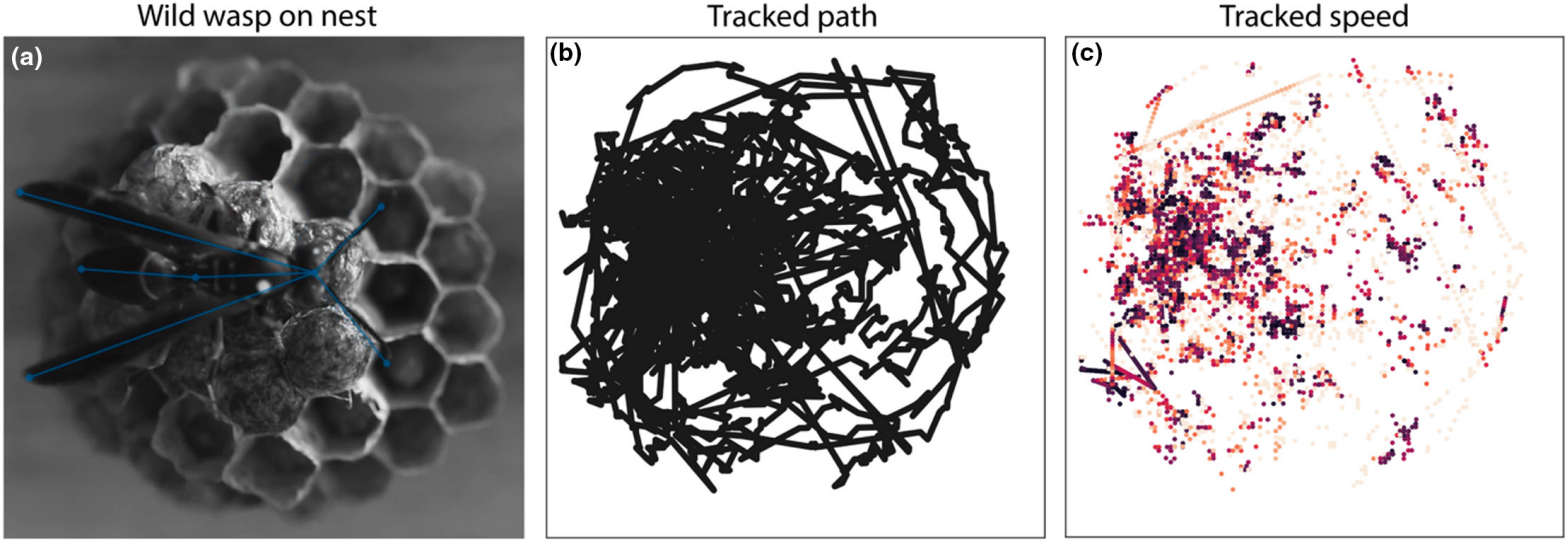
(a) A lone *Polistes fuscatus* foundress on the nest after a simulated intruder encounter. (b) Tracks of the position of the thorax during a 320‐second interval after simulated intrusion. (c) Points designate the position of the thorax and are color‐coded by speed, with lighter colors representing faster movement.

We used computer vision software SLEAP (Pereira et al., 2022) to track seven points on the wasps: antennae tips, head, thorax‐abdomen bridge (propodeum), abdomen tip, and wing tips (Figure 1a; Video 1). In a preliminary analysis, we used DeepLabCut v2.0 to track ten points on the wasps (see Video 2) (Mathis et al., 2018). All analyses reported here are based on data generated using SLEAP, which performed comparatively well. SLEAP was installed on a PC equipped with a GeForce RTX 2080i graphics card. Videos were converted to grayscale and a subset of 20 frames per interval was manually labeled. Raw tracking data and tracked videos are available online (see Data Accessibility statement). We compared the total distance traveled, the mean angle of separation between wing tips (“wing extension angle”), and the mean angle of separation between antennae tips (“antennal separation angle”) before and after stimulus presentations using Wilcoxon signed‐rank tests. Effect sizes were estimated as Wilcoxon effect size *r* using the R package rstatix (version 0.7.2) with method “paired” (Kassambara, 2023). Statistical analyses were performed in R version 4.2.2 (R Core Team, 2019).

## 3 RESULTS

During simulated intruder trials, wasps responded by first antennating the intruder, then aggressively biting, mounting, and stinging the pinned wasp (Videos 1 and 2). These are all stereotyped aggressive behaviors in paper wasps (Lorenzi et al., 1997; Tumulty et al., 2021; West‐Eberhard, 1969). During control trials, wasps investigated the dowel, including antennation and occasional mounting, but did not escalate aggression (Videos 1 and 2). SLEAP successfully tracked body parts in 84 ± 21% (SD) of frames across body parts before and after stimulus presentation (Table 1).

**FIGURE 2:**
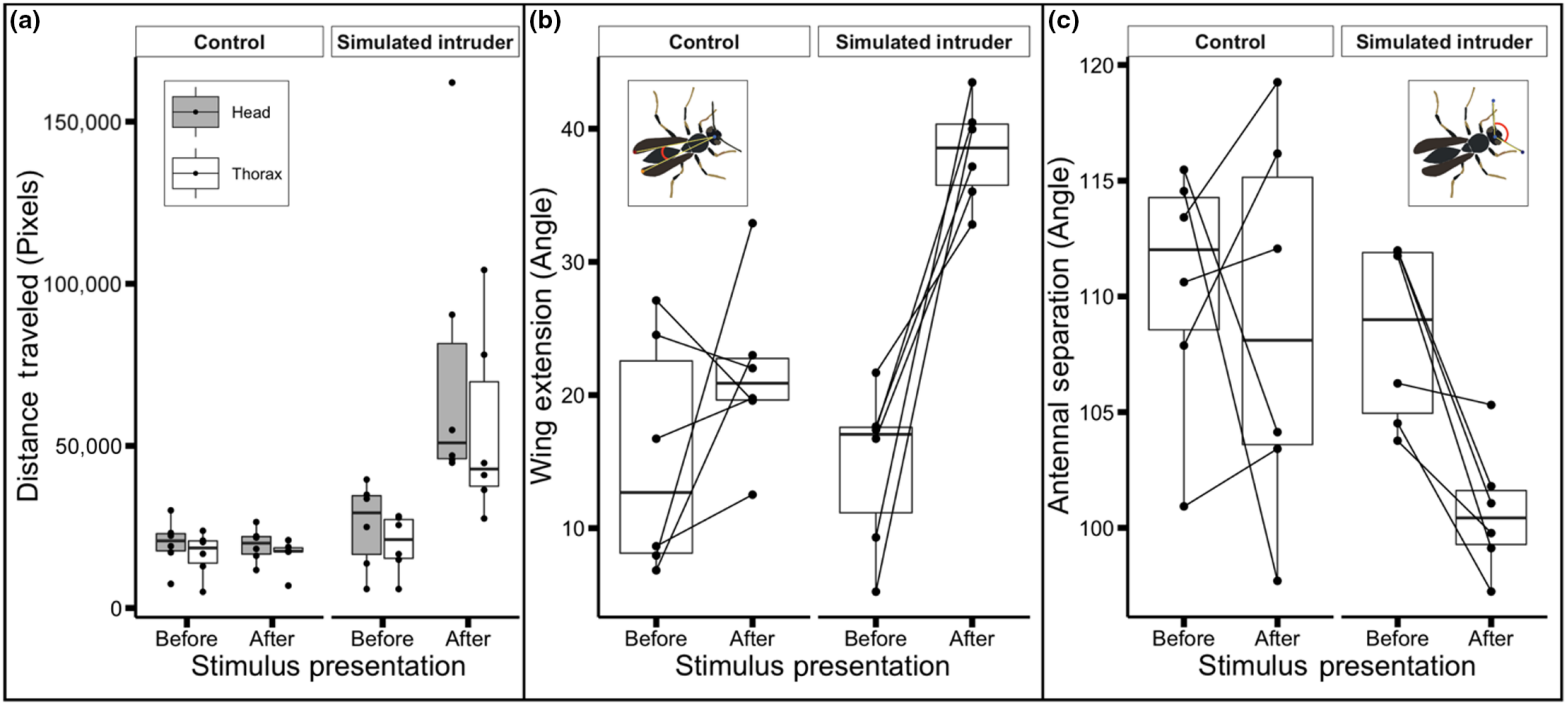
Box and whisker plots display comparisons of measures of movement and posture across trials. (a) Total distance traveled by head (gray) and thorax (white). (b) Wing extension angle. (c) Antennal separation angle.

**FIGURE 3:**
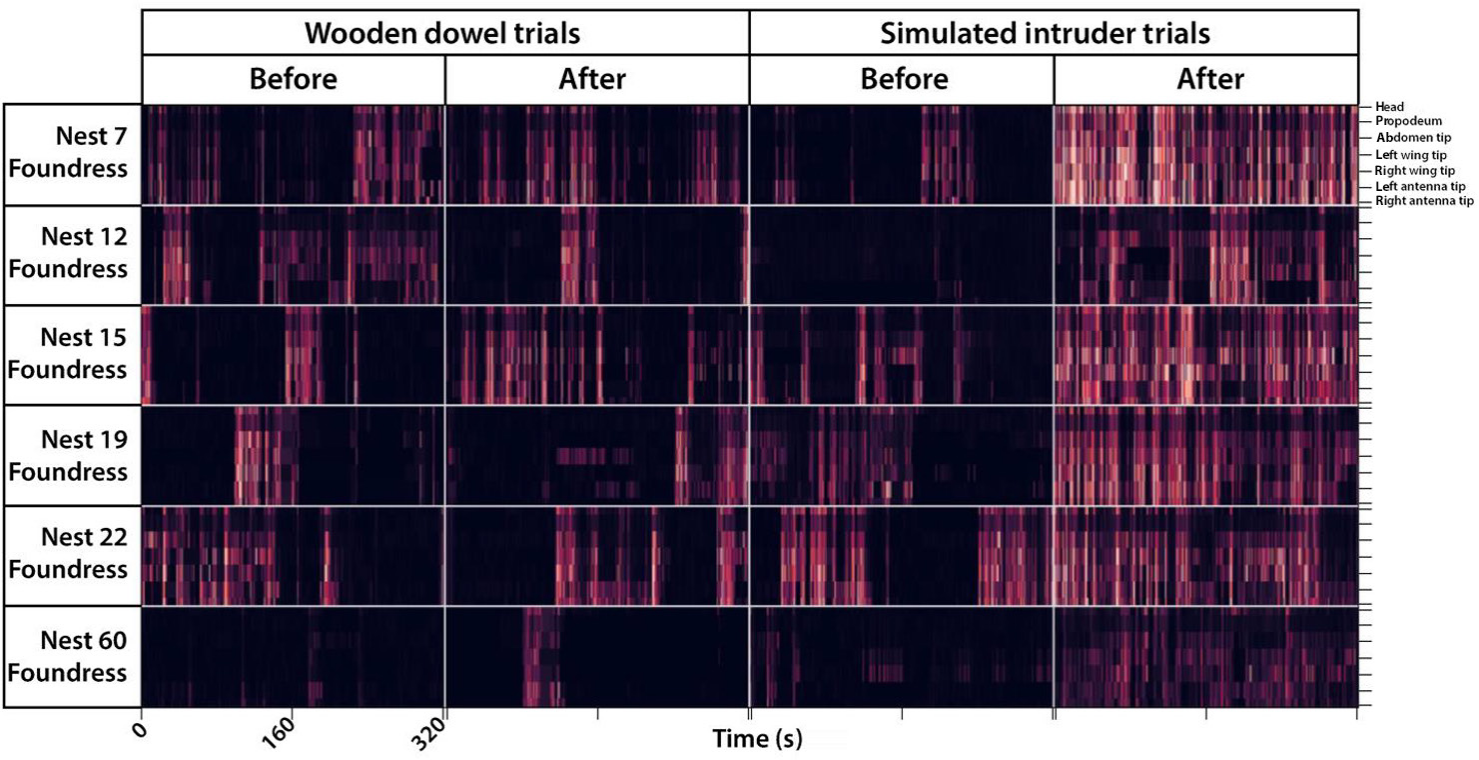
The speeds of seven tracked body parts over time are represented in 24 heatmaps, with lighter colors corresponding to faster speeds. From top to bottom within each heatmap: head, thorax‐abdomen bridge (propodeum), abdomen tip, left wing tip, right wing tip, left antenna tip, right antenna tip.

Simulated intruder encounters caused persistent changes in posture while control experiments did not. Encounters with the simulated intruder caused an increase in the total distance traveled by foundresses, as measured by the distance traveled by the head and thorax after the intruder was removed (head: *V* = 0, *p* = .03125, *r* = .899; thorax: *V* = 0, *p* = .03125, *r* = .899; Figures 2a and 3). Increased movement after the simulated intruder encounter appears to endure throughout the 320‐second observation interval (Figure 3). Encounters with the wooden dowel did not result in sustained increase in movement (head: *V* = 11, *p* = 1, *r* = .0428; thorax: *V* = 12, *p* = .8438, *r* = .128). Foundress wing posture was affected by the simulated intruder. The mean wing extension angle after intruder encounters was significantly greater than before (*V* = 0, *p* = .03125, *r* = .899; Figure 2b). No significant change in mean wing extension angle was observed after wooden dowel presentations (*V* = 5, *p* = .3125, *r* = .471). There was a significant decrease in the mean antennal separation angle after intruder encounters (*V* = 0, *p* = .03125, *r* = .899; Figure 2c). No significant change in mean antennal separation angle was observed after wooden dowel presentations (*V* = 11, *p* = 1, *r* = .0428).

The rapid movement of foundresses during simulated intruder encounters and the presence of a second, pinned wasp precluded successful automated tracking of foundress body posture. However, automated tracking during the dowel presentations was feasible. During the dowel presentation, wasps did not move more than they did before the presentation, based on the total distance traveled by the thorax (*V* = 5, *p* = .3125, *r* = .128). There was a significant increase in wing extension angle during the dowel presentation compared to before (*V* = 0, *p* = .03125, *r* = .899). This increase in wing extension did not persist after the dowel was removed, as reported above.

## 4 DISCUSSION

Encounters with simulated conspecific intruders elicited sustained vigilance‐like behavior in *P. fuscatus* foundresses in the form of increased movement, greater bilateral wing extension, and reduced antennal separation. Natural threats that can induce sustained vigilance behavior in solitary foundresses include intraspecific brood‐robbing and nest usurpation (Gamboa et al., 1992; Kasuya et al., 1980; Sakagami & Fukushima, 1957). The 320‐second lure presentation in our assays likely simulated a worst‐case scenario for foundresses, akin to a prolonged nest usurpation attempt. Three trials in our study were interrupted by natural intruders, highlighting the pervasive nature of conspecific threats to *P. fuscatus* foundresses. While these interruptions reduced our sample size, our statistical analyses were sound given the large estimated effect sizes of the observed changes in foundress movement and posture after intruder encounters (Wilcoxon effect size *r* > .8).

Foundresses that encountered an intruder moved more after the stimulus presentation compared to when they encountered a wooden dowel (Figures 2 and 3). By moving throughout the nest surface, vigilant wasps might be better prepared to defend against an intruder approaching from any direction. Postural changes displayed by vigilant wasps included wing extension and reduced antennal separation (Figure 2). During simulated intruder encounters, foundresses approached the lure with outstretched antennae before reacting aggressively (Video 2). In general, social insects utilize chemical cues to discriminate between nestmates and non‐nestmates (Nunes et al., 2008; Van Zweden & d'Ettorre, 2010). While *P. fuscatus* wasps rely on vision to recognize individual identity, nestmate recognition is mediated by olfaction, possibly facilitated by an expanded repertoire of odorant receptor genes (Legan et al., 2021; Ortiz & Tibbetts, 2020; Tibbetts, 2002). Reduced antennal separation might indicate that wasps are orienting their antennae to detect chemical cues, such as the cuticular hydrocarbon signatures used by social insects to discriminate between nestmates and non‐nestmates (Bruschini et al., 2011; Dani et al., 2001; Gamboa et al., 1986; Nascimento & Nascimento, 2012). Visual cues could also be important in discriminating between nestmates and non‐nestmates in the early phases of the colony cycle, and the absence of nestmates might favor universal rejection (Cini et al., 2019; Reeve, 1989).

Paper wasps are ideal for field‐based automated tracking because their unenveloped nest represents a fixed arena easily recordable by video. In terms of video recording, a drawback to the paper wasp nest architecture is that there is usually space between the nest and the substrate to which it is fixed, so wasps can crawl out of view of the camera behind the nest. While the nest can be treated as two‐dimensional for the purpose of automated tracking, the wasp's body is not always parallel to this plane, leading to difficulties in tracking a wasp perched on the side of the nest. In principle, these challenges could be solved by using multiple cameras to record the nest from different angles, as recently applied in 3‐dimensional tracking in laboratory rodents (Ebbesen & Froemke, 2022; Marshall et al., 2021). Another challenge for automated tracking is the rapid movement of wasps during the simulated intrusions, but cameras with faster frame rates might solve this issue.

In *Polistes*, wing extension and antennal separation might be useful measures for studying how the social environment influences internal state. In the fly, *Drosophila melanogaster*, the reliable associations between unilateral wing extension and courtship, and between bilateral wing extension and aggression, have been useful measures for studying the neural basis of aggression and courtship, especially the roles of P1 neurons in orchestrating persistent internal states causing aggression and courtship (Hoopfer et al., 2015; Zhou et al., 2008). The internal state associated with vigilance‐like behavior in *P. fuscatus* may represent an emotional primitive, as defined by Anderson and Adolphs (2014) as an internal state exhibiting scalability, valence, persistence, and generalization. Regarding scalability, we found preliminary evidence that wing extension can be ordered along a gradient corresponding to low vigilance (before stimulus), medium vigilance (during dowel presentation), and high vigilance (after simulated intruder presentation, demonstrating behavioral persistence). *P. fuscatus* vigilance‐like behavior was associated with aggression towards pinned conspecific intruders, suggesting negative valence. After simulated intruder encounters, changes in behavior were persistent. More work needs to be done to assess the generalization of *P. fuscatus* vigilance behavior, for example by presenting wasps with neutral stimuli after social challenge. Furthermore, future work should incorporate other biologically relevant stimuli to assess the specificity of *P. fuscatus* responses to conspecific intruders.

Increased encounters with non‐nestmate intruders can shift social insect recognition processes to become more exclusive, resulting in recognition errors in the form of increased aggression towards nestmates (Couvillon et al., 2008; Downs & Ratnieks, 2000; Mora‐Kepfer, 2014; Scharf et al., 2020; Starks et al., 1998). From the perspective of signal detection theory, individual vigilance behavior could be mechanistically related to acceptance threshold shifts. If persistent vigilance and acceptance threshold shift are coupled, then there will be more aggression towards nestmates following intruder encounters. Alternatively, persistent vigilance might have effects on recognition independent of acceptance threshold shifts. For example, persistent vigilance might accompany increased investment in accurate recognition (Sheehan & Reeve, 2020). Evidence supporting this alternative may be found in the carpenter ant, where exposure to alarm pheromone increased accuracy of both nestmate acceptance and non‐nestmate rejection (Rossi et al., 2019). Persistent vigilance might therefore increase recognition accuracy, while the acceptance threshold is shifted depending on non‐nestmate encounter rates (Reeve, 1989; Wiley, 2013).

Because this was the first research project to apply automated tracking in the study of wild paper wasp posture, we studied lone foundress nests to ensure automated tracking would be feasible. The automated tracking results from our study showed that individual wasp movement, wing separation angle, and antennae separation angle were markedly different after presentation with a pinned conspecific, and these changes persisted after the threat was removed. While these results support the hypothesis that social challenge results in heightened vigilance, future experiments should incorporate more stimuli to determine whether the responses are specific to conspecific challenge. Future work should also examine measures of movement and posture in multiple foundress nests, or in nests with multiple workers present, in order to explore how individual wasp vigilance behavior relates to shifts in nestmate recognition processes.

## AUTHOR CONTRIBUTIONS

**Andrew W. Legan:** Conceptualization (lead); data curation (lead); formal analysis (lead); funding acquisition (equal); investigation (lead); methodology (equal); project administration (lead); resources (equal); software (supporting); supervision (equal); validation (lead); visualization (lead); writing – original draft (lead); writing – review and editing (lead). **Caleb C. Vogt:** Conceptualization (supporting); data curation (supporting); formal analysis (equal); funding acquisition (supporting); investigation (equal); methodology (equal); project administration (supporting); resources (equal); software (lead); supervision (supporting); validation (equal); visualization (supporting); writing – original draft (supporting); writing – review and editing (supporting). **Michael J. Sheehan:** Conceptualization (supporting); funding acquisition (equal); methodology (equal); project administration (supporting); resources (lead); supervision (lead); writing – original draft (supporting); writing – review and editing (supporting).

## FUNDING INFORMATION

The work was funded by the National Science Foundation, Cornell University, the National Institutes of Health, and the North American Section of the International Union for the Study of Social Insects.

## CONFLICT OF INTEREST STATEMENT

The authors have no competing financial or non‐financial interests that are directly or indirectly related to this work.

## Data Availability

Videos are available for open access in CERN's Zenodo repository (Legan, 2022; https://doi.org/10.5281/zenodo.6582229). Raw data from automated tracking are available in the Figshare repository (Legan et al., 2023; https://doi.org/10.6084/m9.figshare.22706437).
